# The experiences of receiving a diagnosis of attention deficit hyperactivity disorder during adulthood in Japan: a qualitative study

**DOI:** 10.1186/s12888-020-02774-y

**Published:** 2020-07-16

**Authors:** Yumi Aoki, Takashi Tsuboi, Takehiko Furuno, Koichiro Watanabe, Mami Kayama

**Affiliations:** 1 Psychiatric and Mental Health Nursing, Graduate School of Nursing, St. Luke’s International University, 10-1 Akashi-cho, Chuo-ku, Tokyo, 104-0044 Japan; 2 Department of Neuropsychiatry, School of Medicine, Kyorin University, 6-20-2 Shinkawa, Mitaka-shi, Tokyo, 181-8611 Japan; 3 Department of Psychiatry, Tokyo Medical Center, 2-5-1 Higashigaoka, Meguro-ku, Tokyo, 152-8902 Japan

**Keywords:** Adults, Attention deficit hyperactivity disorder, Developmental disabilities, Qualitative research

## Abstract

**Background:**

Although the number of adults with attention deficit hyperactivity disorder (ADHD) has increased considerably in recent years, there are few qualitative investigations of the experiences of adults with adult-diagnosed ADHD in Japan. This study aimed to explore in depth the diagnosis-related experiences and needs of such adults.

**Methods:**

Participants were 12 psychiatric outpatients aged 23–55 years diagnosed with ADHD during adulthood. Individual semi-structured interviews were conducted to examine participants’ experiences of receiving, and subsequently coping with, an ADHD diagnosis. A thematic analysis of the interview data was performed.

**Results:**

Six themes emerged: difficulties in accepting the diagnosis, interest in ADHD, feelings of relief, identity concerns, dealing with symptoms, and acceptance of ADHD. Despite initial negative reactions, participants were willing to learn about the disorder, spending time seeking ADHD-related information and sharing it with loved ones. Participants felt relieved after the diagnosis, as they realized why they had experienced long-term problems and incorrect labeling. However, participants also had identity concerns. They gradually began to accept their ADHD symptoms and deal with them better.

**Conclusions:**

The results suggest that, when treating individuals with adult-diagnosed ADHD, it is important to promote self-understanding and reduce negative attitudes toward ADHD; to provide appropriate, brief, evidence-based information about ADHD; and to give individuals sufficient time to think about their ADHD symptoms, how they have affected their daily lives, and how to cope with them in the future.

## Background

Attention deficit hyperactivity disorder (ADHD) is a chronic neurodevelopmental disorder characterized by the following behaviour-related symptoms: inattention, hyperactivity, and impulsivity [1]. Although ADHD was once considered to be restricted to children, research over the last few decades has established that most children with ADHD retain at least some symptoms into adulthood [2]. The prevalence of adult ADHD is estimated as 1.65% in Japan [3] and 1.2 to 3.2% [2, 4] worldwide. Thus, a substantial number of adults have ADHD symptoms. In fact, the number of adults with neurodevelopmental disorders has increased considerably in Japan: in 2017, there were 420,000 outpatients with neurodevelopmental disorders, including ADHD; 133% of the previous year’s figure [5].

Three types of pharmacotherapy have been approved for adults with ADHD in Japan: atomoxetine, which has been available since 2012, methylphenidate extended-release tablets since 2013, and guanfacine since 2019. However, unlike other countries with existing clinical guidelines for adults with ADHD, such as the UK, Canada, and Australia [6–8], there are currently no treatment guidelines for adults with ADHD in Japan. Thus, individual clinicians are responsible for developing intervention plans for adult ADHD. The first step of developing a more comprehensive approach to adults newly diagnosed with ADHD is to explore their experiences related to ADHD diagnosis.

Thus far, several qualitative studies to explore the experiences of adults with ADHD have been conducted in European countries. Matheson et al. revealed the burdens experienced by adults with ADHD such as difficulties to access services and barriers to continue treatment [9]. Schrevel et al. suggested that their burdens were strongly interrelated to the social environment, which led to a persistent low self-image [10]. On the other hand, Sedgwick et al. examined the positive aspects of adult ADHD such as cognitive dynamism, energy, divergent thinking, hyperfocus, nonconformist, adventurousness, and self-acceptance and sublimation [11]. Furthermore, some researchers explored diagnosis-related experiences. Hansson Halleröd et al. reported that receiving an ADHD diagnosis confronted both positive aspects (e.g., I can help myself) and negative aspects (e.g., “The diagnosis causes decreased value”) [12]. Young et al. suggested six psychological stages of receiving ADHD diagnosis: relief and elation, confusion and emotional turmoil, anger, sadness and grief, anxiety, and accommodation and acceptance [13]. Even for older ages, it is not unusual to be diagnosed with ADHD. Henry and Jones conducted interviews with individuals who were diagnosed with ADHD after the age of 60, which suggested that they were gradually finding creative solutions to their attention deficit problems [14].

Compared with qualitative studies of adults with ADHD in European countries as described above, to the best of our knowledge, there are no qualitative descriptive studies exploring the experiences of adults with adult-diagnosed ADHD in Japan. This study aimed to explore and better understand the diagnosis-related experiences and needs of this group.

## Methods

### Study design

A qualitative approach using semi-structured interviews was used to enable a detailed, in-depth exploration of individuals’ experiences and needs. Qualitative techniques are particularly useful in improving knowledge of poorly understood areas of health care [15].

### Participants

Participants due to attend follow-up appointments at three psychiatric services in Tokyo during the research period of June 2016 to December 2016 were invited to participate in the study. The inclusion criteria were outpatients (i) aged 20 years or older and (ii) who had received a diagnosis of ADHD during adulthood (aged 17 years or over). The exclusion criteria were (i) not fluent in Japanese, (ii) refusal to provide written informed consent, and (iii) unable to participate in the interview owing to severe symptoms. The researcher, who was totally independent from the psychiatric services, recruited the participants. A purposive sampling strategy was used. Outpatients who regularly visited the three psychiatric services were approached following the inclusion and exclusion criteria described above. Immediately after the routine appointments, the researcher fully explained the aim and overview of this study to potential participants using a written document. Participation was voluntary. Once the potential participants received the explanation, they had sufficient time outside the services to consider whether or not to agree to take part in. Thereafter, consent was taken in the next appointment. A gift voucher of 1000 Japanese yen was given to the person who participated in this study.

Participants were 12 individuals diagnosed with ADHD during adulthood. The mean age at the interview was 36.5 years and the mean age when ADHD was diagnosed was 33.5 years. Table 1 shows demographic characteristics of the 12 participants.

**Table 1:** Demographic characteristics of study participants

ID	Age (years)	Years since ADHD diagnosis	Sex	Occupation	Comorbidity	Medication treatment
A	20–24	2	F	college student	depression	–
B	20–24	1	F	full-time job	panic disorder	anti-anxiety agent
C	25–29	1	F	freelance	depression, eating disorder, sleeping disorder, panic disorder	ADHD medication
D	25–29	9	M	freelance	depression	ADHD medication
E	25–29	4	F	part-time job	depression, panic disorder	anti-depressant
F	55–59	1	M	full-time job	sleeping disorder	–
G	50–54	2	M	full-time job	–	ADHD medication
H	50–54	2	M	none	depression	ADHD medication
I	25–29	5	F	full-time job	–	ADHD medication
J	50–54	3	M	full-time job	–	ADHD medication
K	45–49	0	F	freelance	–	–
L	20–24	0	M	college student	–	ADHD medication

### Data collection

The researcher (YA), who had received prior training in qualitative methodology, conducted the semi-structured interviews in Japanese. YA is an assistant professor in the Department of Psychiatric Nursing with experience in psychiatric care, and has published qualitative research on experiences of people with mental health conditions in international journals. YA is a native Japanese speaker and familiar with Japanese culture. Face-to-face interviews were conducted in the consultation room as a private space immediately following their routine appointments. An interview guide was used containing the following questions:

- How did you feel when ADHD was first diagnosed?
- What did you do after the diagnosis?
- Did your feelings about the diagnosis change with time; if so, in what way?

The full interview guide is available in Additional file 1. The socio-demographics such as age, years since ADHD diagnosis, sex, occupation, commodity, and current medication treatment were also collected. The interviewer had not been involved in any previous assessment and/or ongoing treatment of participants. Each interview was recorded and subsequently fully transcribed by the researcher.

### Analysis

A thematic data analysis, following the six phases proposed by Braun and Clarke [16], was used. The first phase involved familiarization with the data. The recorded interviews were transcribed verbatim. Each interview was read several times to identify ideas, patterns, and meanings in each interview transcript. The second phase comprised the generation of initial codes from the data. In this phase, significant examples of codes were extracted (using quotes from participants) and then thoroughly coded. The third phase involved searching for themes. Similar codes were assembled, and subthemes developed. Then, similar subthemes were combined, and initial themes were generated. In the fourth phase, these themes were reviewed. This phase involved two levels of reviewing and refining of the themes. In level one, the themes were reviewed at the level of the extracted coded data. In level two, the entire data set was reviewed, to consider the validity of individual themes in relation to the data set. The fifth phase comprised defining and naming themes. In this phase, a thematic map of the data was developed. The entire analysis process was reviewed to refine the features of each code and the story that the data represented. Each theme was named and defined. The sixth phase involved producing the report. The researcher (YA) conducted the analytical work independently but it was audited by a co-researcher (MK), who is an expert in qualitative analysis to establish trustworthiness. All analyses were conducted in Japanese.

## Results

Six themes and nine subthemes describing the experience of receiving a diagnosis of ADHD during adulthood were derived from the interview data. The six themes were as follows: difficulties in accepting the diagnosis, interest in ADHD, feelings of relief, identity concerns, dealing with symptoms, and acceptance of ADHD.

The following storyline of participants’ experiences emerged from the analysis. Immediately following diagnosis with ADHD, participants were upset and found it difficult to accept the diagnosis. Despite this initial negative reaction, however, participants were willing to learn about the disorder, spending time seeking information about ADHD and sharing it with loved ones. They experienced some relief through information seeking and sharing, as they realized why they had been struggling with problems and had labelled themselves incorrectly for a long time. At the same time, the more they learned about ADHD, the more confused they felt about their identity. Although participants experienced mental turmoil, they gradually became better at dealing with ADHD symptoms by acquiring coping skills, taking ADHD medication, and adjusting their work environment. Participants also became more accepting of the inevitability of the ADHD symptoms.

The themes are described below with illustrative quotes from the participants.

### Theme 1: difficulties in accepting the diagnosis

#### Lack of knowledge

Immediately after ADHD diagnosis, many participants became upset and felt unable to accept the diagnosis. Several reasons for this were expressed. First, participants did not have sufficient knowledge about ADHD. Some participants felt confused and unclear about what they should do, as they had never heard of ADHD. Although some participants had heard of ADHD, some interviewees thought that it only occurred in childhood and did not know that ADHD symptoms could continue into adulthood.

*“My ADHD diagnosis was quite sudden and I did not know what was what anymore, because I did not know anything about ADHD.” (B, female aged 20–24).*

*“I thought ADHD was a disease of young boys! Why me? You must be joking!” (A, female aged 20–24).*

#### Self-stigma

Self-stigma about developmental disorders made it difficult for participants to accept the ADHD diagnosis. Even those not familiar with ADHD had heard of developmental disorder. Many participants had negative attitudes and biased views toward developmental disorders. Once they had been diagnosed with ADHD and realized that it was a developmental disorder, their latent stigmas started to emerge and reflect back as self-stigmas.

“*I disliked the word ‘developmental disorder’. It seemed to indicate lack of development and looking stupid.” (C, female aged 25–29).*

*“[People with developmental disorder] seem to be odd. I was shocked and felt disgusted that I was also one of those oddballs.” (I, female aged 25–29).*

### Theme 2: interest in ADHD

#### Information seeking

All participants attempted to obtain information about ADHD and its treatments after ADHD diagnosis. Participants picked up pamphlets from the waiting room in the service clinic, purchased books about ADHD, and looked up the term “ADHD” on the Internet at home. They were interested in ADHD and actively sought information about the disorder. Most participants wanted to learn what ADHD was and how to cope with it.

“*Like with my asthma, I carefully looked up ADHD because I was worried about being able to understand the situation if something bad happened.” (B, female aged 20–24).*

*“I really spent time looking up ADHD on the Internet.” (J, male aged 50–54).*

#### Information sharing

During information seeking, many participants were willing to share information about the disorder and its treatment with their loved ones. The family members of some participants also actively gathered information about ADHD so they could discuss the disorder and its treatments. Some participants talked to friends or acquaintances who were receiving psychiatric treatment. All participants sought to deepen their knowledge about ADHD and share it with others close to them.

*“I talked to my mother a lot about my ADHD and revealed it to my boss, too.” (B, female aged 20–24).*

*“I asked my friend, who is receiving medication for psychiatric problems, ‘What is your experience like?’” (K, female aged 45–49).*

### Theme 3: feelings of relief

#### Realizing why they had experienced long-term problems

Along with increased knowledge about the characteristics and symptoms of ADHD, participants gradually began to understand that they had experienced ADHD symptoms since childhood. Many felt relieved when they realized that their symptoms were at the root of problems and difficulties they had had or sensed. They became aware of why they had experienced long-term problems throughout their lives.

*“Something unknown and unidentified, which I have had for a long time, has been cleared up. I feel so much better knowing about ADHD.” (G, male aged 50–54).*

*“It was just like all the precursors of my story had converged.” (L, male aged 20–24).*

#### Realization of their history of incorrect labelling

Many participants had felt different from others for a long time. Participants’ friends and social acquaintances had often cautioned them about their behaviours. Participants had then been identified (and had labelled themselves) as inferior to their classmates.

*“I was relieved, as I knew that my label of a sloppy person or a lazy person was inappropriate.” (K, female aged 45–49).*

Moreover, those who had sought psychiatric treatment previously had been diagnosed with different mental disorders. One participant explained that she had been diagnosed with other diseases like schizophrenia, bipolar disorder, panic disorder, and sleeping disorder before the ADHD diagnosis. She now realized that all her previous disorders had been caused by ADHD symptoms, although each disease had been treated separately.

*“Although I had been dealing with my difficulties as indications of other diseases for a long while, all my past experiences could be explained by ADHD.” (C, female aged 25–29).*

### Theme 4: identity concerns

Despite feeling emotional relief, participants experienced doubt or confusion about their identity. As they realized that their ADHD symptoms accounted for their traits and behaviours toward others, participants began to ask themselves “Who am I really?” One participant felt that the impression of himself he had had since childhood had been shattered, because the ADHD symptoms were the real cause of his character. These concerns about self-identity generated anxiety, as participants felt that ADHD symptoms defined, and perhaps dominated, their lives. Another participant stated “I am myself,” even after his ADHD diagnosis. He could not imagine himself without the ADHD symptoms. He now perceived his traits as ADHD symptoms, although they had always been part of him.

*“Well then, ‘Who am I really?’ I thought.” (D, male aged 25–29).*

*“The more I learned about ADHD, the more I doubted who I was … Are my assumptions about myself so far, all wrong? I started to feel anxious.” (E, female aged 25–29).*

### Theme 5: dealing with symptoms

#### Acquiring coping skills

Gradually, many participants acquired their own coping behaviours and skills to deal with ADHD symptoms. In seeking information about ADHD and how to cope with it, they looked back over their lifestyle and acquired skills and considered how these expressed their symptoms. For instance, participants reported the following coping skills: taking notes, even about “small stuff”; writing down any ideas that “enter my head” before speaking; listening to others until they have finished speaking; and setting an alarm 15 min before they had to leave the house as a reminder. The ADHD diagnosis gave participants the opportunity to acquire more relevant and useful coping skills.

*“I knew getting distracted easily was one of the symptoms and I tried to deal with it.” (K, female aged 45–49).*

#### Taking medication

Taking medication was another way of dealing with ADHD symptoms and some participants felt quite positive about taking medications. Most were willing to obtain information about appropriate medication from websites, books, and other people (such as family members and friends) in addition to receiving information from their psychiatrists. One participant reported that the psychiatrist’s explanation had not been very clear and so he found it difficult to understand the positive and negative aspects of medication treatment even though he was willing to receive it.

*“Depending on circumstances, it is better to have no ADHD symptoms. So, I started to take the ADHD medication as a tool to deal with my ADHD symptoms.” (L, male aged 20–24).*

#### Adjusting the work environment

Participants felt that it was important not only to learn new coping skills for daily life or to control symptoms by medication, but also to adjust their working environment to suit their talents and symptoms. The symptoms of ADHD can be influenced, for better and worse, by the environment or the attitudes of others; therefore, some participants disclosed their ADHD diagnosis to their boss and colleagues at work and accepted the accommodations offered to them for their symptoms. Participants who had consulted a psychiatrist about maladaptation to the workplace found it easy to disclose their ADHD symptoms to others.

*“I decided I was not going to deal with it just by myself anymore, and I let my boss know about my ADHD. My boss reminds me about due dates and informs me about my daily schedule.” (J, male aged 50–54).*

### Theme 6: acceptance of ADHD

Participants finally recognized that their ADHD diagnosis was inevitable and undeniable. Prejudices and negative attitudes toward ADHD gradually disappeared and participants became more willing to accept the diagnosis. Many participants had previously expressed negative judgments about their problematic behaviours, such as agitated behaviour in public, incoherent or disruptive talking in front of others, and being untidy. Subsequently, they stopped blaming themselves and struggling with such symptoms. Participants started to move beyond their negativity and began to accept ADHD.

*“My prejudice about ADHD has decreased over the last year.” (C, female aged 25–29).*

*“It is a disease, unavoidable, not my fault, and not just me … Knowing it is a brain disease made me stop resisting it.” (K, female 45–49).*

**Fig. 1:**
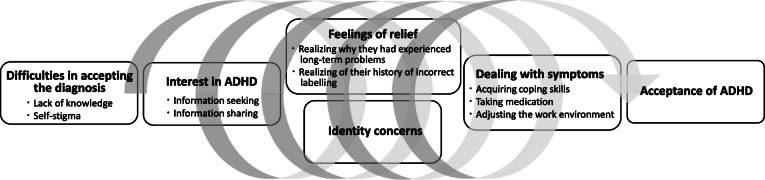
The diagram of the experiences of receiving ADHD diagnosis during adulthood

Figure 1 illustrates the relationship between these themes, which describe the experience of receiving a diagnosis of ADHD during adulthood.

The spiral indicates that the process was not always straightforward: participants sometimes felt they were moving forward, but at other times felt they were going backwards before they reached an acceptance of their condition.

## Discussion

To our knowledge, this is the first study to investigate diagnosis-related experiences of adults diagnosed with ADHD during adulthood in Japan.

### Similarities to previous research

In addition to feeling emotional relief, participants also experienced identity confusion, which is in accord with findings from some European studies. Hansson Halleröd et al. found that participants who received an ADHD diagnosis during adulthood described how their diagnosis explained a previously inexplicable life history, but also caused devaluation and identity concerns [12]. Young et al. also reported that adults with ADHD experienced turmoil and confusion as they reviewed and reframed their past experiences in light of their diagnosis and acquired a new understanding of themselves [13]. Davies and Horton-Salway also explained that a diagnosis of adult ADHD raised identity issues through the lens of discursive psychology [17]. People diagnosed with ADHD during childhood develop their characters and identities while learning about ADHD symptoms and acquiring coping skills with support from others. ADHD diagnosis during adulthood requires patients to accept symptoms that have been concealed or unrecognized during childhood and are now more obvious owing to environmental circumstances or social relationships. Previous research has also identified a gradual acceptance of the condition. Young et al. found that patients gradually accept ADHD symptoms after experiencing confusion and emotional turmoil [13]. Stenner et al. revealed that troubled pasts are reconstructed from the viewpoint of new identity that offers adults with ADHD the potential for a more enabling and positive future [18]. Thus, a transformed version of the self is achieved after medical intervention and ADHD diagnosis [19].

### Notification of ADHD diagnosis

As participants reflected back on their lives and recognized that their ADHD symptoms now made complete sense, they felt relieved. This suggests that if a psychiatrist identifies ADHD symptoms, it is important that individuals are informed immediately although notification of an ADHD diagnosis may not necessarily occur in a psychiatric setting in Japan [20]. It is important that caregivers provide adults with ADHD with appropriate evidence-based information about ADHD. Biological and objective descriptions related to brain function are also useful because biological explanation is often preferred to manage the spoiled identities in clinical contexts [17]. Furthermore, it is crucial that adults newly diagnosed with ADHD have sufficient time to deliberate about their ADHD symptoms and how they may have affected their daily lives.

### Approaches for stigma relief

Immediately after receiving the ADHD diagnosis, participants thought about negative images and stigmatizing attitudes toward this developmental disorder. This seems unique to this study, as previous research has not reported such a finding. Several studies demonstrated that ADHD is strongly associated with stigmatization [19, 21]. Weiner and colleagues concluded that stigma deriving from behavioural deviance is more pronounced than stigma associated with physical impairment, because of the stronger association between uncontrollability and norm-violating behaviour in the general public [22]. According to Burch, ADHD in adulthood is more likely than ADHD in childhood to be associated with misperceptions and confusion, and a large number of laypeople and professionals lack disorder-related knowledge [23]. Therefore, when describing or explaining about adult ADHD to individuals diagnosed with ADHD during adulthood, it is crucial that caregivers use expressions that promote accurate knowledge and eliminate prejudice. The International Classification of Functioning, Disability and Health defines disabilities not as fixed handicaps but as a lack of proper functioning influenced by social or environmental conditions [24]. When explaining adult ADHD, it is important to emphasize that ADHD symptoms are not fixed, but fluctuate. Thus, they are changeable and can be influenced by a person’s coping skills or environmental conditions.

### Limitations

This study had several limitations. First, the sample may have been biased as participants were recruited from a population that regularly had psychiatric consultations. Second, the period between the ADHD diagnosis and the interview varied between individuals from less than 1 year to nearly 10 years (see Table 1). Therefore, some participants might not have remembered all the experiences at the time of their ADHD diagnosis, and focused on remarkable experiences. Third, we used a qualitative approach to examine in detail the experiences of a small number of participants. Future studies using a mixed-methods approach with outcome measures might be useful. However, the aim was to clarify and better understand individuals’ experiences of an ADHD diagnosis. This aim was met.

## Conclusion

Adults diagnosed with ADHD during adulthood in Japan experienced difficulties in accepting their diagnosis, interest in ADHD, feelings of relief, identity concerns, dealing with symptoms, and acceptance of their condition. When treating adult-diagnosed individuals, it is necessary to supplement the ADHD diagnosis with appropriate evidence-based information; promote self-understanding and reduce stigmatized attitudes toward ADHD; and provide individuals with sufficient time to think about ADHD symptoms, to consider how they may have affected daily life, and to develop ways to cope with ADHD in the future.

## Supplementary information

**Additional file 1.** Interview Guide.

## Data Availability

The interview data of the current study are available from the corresponding author on reasonable request.

## Abbreviation

ADHD: Attention deficit hyperactive disorder
