# Supplementary material for: The experiences of receiving a diagnosis of attention deficit hyperactivity disorder during adulthood in Japan: a qualitative study
Source: BMC Psychiatry. 2020 Jul 16;20:373. doi: 10.1186/s12888-020-02774-y (PMC7366299; doi:10.1186/s12888-020-02774-y)
Supplement: Supplementary file 1 — Additional file 1. Interview Guide. [file 12888_2020_2774_MOESM1_ESM.pdf]

Interview Guide

1) Before diagnosis

・How did you spend your life before you were diagnosed with ADHD?

・What made you come to see a doctor? (situation, triggers, troubles in daily life)

・How did you feel about your condition before coming?

・Did you have any advice from your family or people around you at work (school) before coming?

2) When you were diagnosed with ADHD

・What were your initial thoughts? (good things and bad things)

・What kind of explanation did you receive from your doctor?

・How did you feel when you heard the explanation from your doctor?

・How did you feel about your symptoms?

・Are there any explanations other than from your doctor; if so, how were they and how did you feel when you heard about them?

3) After ADHD diagnosis

・What did you do after your diagnosis?

・Did your feelings about the diagnosis change with time; if so, in what way?

・Since your ADHD diagnosis, are there any changes at work/school?

・Since your ADHD diagnosis, are there any changes in your life at home?

・What do you think will your life be in the future?
